# Progression to AIDS in SIV-Infected Rhesus Macaques is Associated with Distinct *KIR* and *MHC class I* Polymorphisms and NK Cell Dysfunction

**DOI:** 10.3389/fimmu.2014.00600

**Published:** 2014-11-28

**Authors:** Christina Albrecht, Dörthe Malzahn, Markus Brameier, Meike Hermes, Aftab A. Ansari, Lutz Walter

**Affiliations:** ^1^Primate Genetics Laboratory, German Primate Center, Leibniz-Institute for Primate Research, Göttingen, Germany; ^2^Department of Genetic Epidemiology, University Medical Center, Georg-August-University, Göttingen, Germany; ^3^Department of Pathology and Laboratory Medicine, Emory University School of Medicine, Atlanta, GA, USA

**Keywords:** virus infection, killer cell immunoglobulin-like receptors, MHC class I ligands, genetic polymorphism, epistasis, rhesus macaque SIV infection model, AIDS

## Abstract

Killer cell immunoglobulin-like receptors (KIR) regulate the activity of natural killer (NK) cells and have been shown to be associated with susceptibility to a number of human infectious diseases. Here, we analyzed NK cell function and genetic associations in a cohort of 52 rhesus macaques experimentally infected with SIVmac and subsequently stratified into high viral load (HVL) and low viral load (LVL) plasma viral loads at set point. This stratification coincided with fast (HVL) and slow (LVL) disease progression indicated by the disease course and critical clinical parameters including CD4+ T cell counts. HVL animals revealed sustained proliferation of NK cells but distinct loss of peripheral blood NK cell numbers and lytic function. Genetic analyses revealed that *KIR* genes *3DL05*, *3DS05*, and *3DL10* as well as *3DSW08, 3DLW03*, and *3DSW09* are correlated, most likely due to underlying haplotypes. SIV-infection outcome associated with presence of transcripts for two inhibitory *KIR* genes (*KIR3DL02*, *KIR3DL10*) and three activating *KIR* genes (*KIR3DSW08*, *KIR3DS02*, *KIR3DS05*). Presence of *KIR3DL02* and *KIR3DSW08* was associated with LVL outcome, whereas presence of *KIR3DS02* was associated with HVL outcome. Furthermore, we identified epistasis between *KIR* and *MHC class I* alleles as the transcript presence of the correlated genes *KIR3DL05*, *KIR3DS05*, and *KIR3DL10* increased HVL risk when Mamu-B*012 transcripts were also present or when Mamu-A1*001 transcripts were absent. These genetic associations were mirrored by changes in the numbers, the level of proliferation, and lytic capabilities of NK cells as well as overall survival time and gastro-intestinal tissue viral load.

## Introduction

Natural killer (NK) cells are large bone marrow-derived granular lymphocytes that perform essential functions in the innate immune response against various pathogens and in the priming of adaptive immunity. The main effector functions of NK cells are target cell killing and cytokine release, which are regulated via integration of signals derived from stimulatory and inhibitory receptors expressed on the cell surface of these lymphocytes. NK cells are important effector cells to combat infection with viruses (1), including human and simian immunodeficiency viruses (SIVs) (2–4).

Killer cell immunoglobulin-like receptors (KIR) are crucial regulators of NK cell activity and exhibit considerable allelic polymorphism and copy-number variations in humans and higher primates (5, 6). Epidemiological studies of human immunodeficiency virus 1 (HIV-1) infection indicate that a combination of HLA-Bw4-80I with either KIR3DL1 or KIR3DS1 is associated with delayed progression to AIDS (3, 7–9). Differences in expression levels of KIR3DL1 allotypes correlate with different functional capacities of NK cells (10), and KIR3DL1 allotypes expressed at high levels are associated with delayed progression to AIDS (11). While the role of inhibitory KIR3DL1 in the regulation of NK cell activity toward HIV-1-infected cells is plausible, the molecular basis underlying the epistasis of *KIR3DS1* with *HLA-Bw4-80I* is unknown and any demonstration of physical interaction between KIR3DS1 and HLA-Bw4 proteins has so far been unsuccessful. Nevertheless, KIR3DS1-positive NK cells were reported to inhibit HIV-1 replication more effectively on target cells expressing HLA-Bw4-80I compared to KIR3DS1-negative NK cells (12). In addition, both KIR3DS1- and KIR3DL1-positive NK cell proportions are elevated during acute HIV-1 infection in the presence of HLA-Bw4-80I molecules (13). A recent paper by Song and colleagues (9) confirmed these findings and demonstrated the impact of the strength of educational signals obtained via the KIR3DL1 receptor and an important role of CC-chemokine release to inhibit HIV-1 replication. Further support for the impact of NK cells on HIV-1 infection was documented in studies of HIV-1 peptide escape mutations that resulted in stronger interaction of the inhibitory KIR2DL2 with HLA-C1 presenting mutated peptides (14).

Simian immunodeficiency virus (SIV)-infected rhesus macaques represent an established and important animal model to study the mechanisms of HIV infection. SIV-induced syndromes and the temporally diverse progression to AIDS of SIV-infected rhesus macaques are remarkably similar to human HIV-1 infection with variable disease outcome ranging from spontaneous control of plasma viremia (elite controllers, about 5–20% of infected individuals) to uncontrolled viremia and rapid disease course (fast progressors, about 5–10%). These polarized clinical outcomes during persistent viremia in experimentally infected rhesus macaques suggest genetic variability may play a critical role in SIV containment. Rhesus macaque *KIR* genes are considered as candidate genes that contribute to different clinical outcomes as they are at least as diverse as human *KIR* genes (15–18). Indeed, in SIV-infected rhesus macaques, copy number variations (CNV) of the inhibitory *KIR3DL05* gene were shown to be associated with fast progression to AIDS, whereas CNVs of activating *KIR3DS* genes were associated with control of virus replication (19, 20). However, it is clear that the studies of the beneficial or detrimental combinations of *KIR* and *MHC class I* polymorphisms are in their infancy and much has yet to be learnt about these associations. Further, it is unclear whether changes in the level of expression of KIRs occur during the course of SIV infection and whether these changes contribute to disease pathogenesis.

Herein, we demonstrate that NK cells in SIV-infected rhesus macaques with rapid disease progression and high viral load (HVL) show sustained proliferation but less lytic activity, followed by a dramatic depletion of NK cells from the peripheral blood. This functional impairment is accompanied by changes of transcription of five *KIR* genes (inhibitory: *KIR3DL02*, *KIR3DL10*; activating: *KIR3DSW08*, *KIR3DS02*, *KIR3DS05*). Presence of the correlated *KIR3DL05*, *KIR3DS05*, and *KIR3DL10*, genes combined with presence of Mamu-B*012 or with lack of Mamu-A1*001 was more frequently found in animals rapidly progressing to disease, and animals with such *KIR*/*MHC class I* combinations displayed higher gastro-intestinal tissue (GIT) viral loads and shorter survival times.

## Materials and Methods

### Ethical statement

Juvenile to adult male rhesus macaques (*Macaca mulatta*) of Indian origin were used for the studies reported herein. All animals were born and maintained at the Yerkes National Primate Research Center of Emory University (Atlanta, GA, USA) in accordance with the regulations of the Committee on the Care and Use of Laboratory Animal Resources, National Research Council and the Department of Health and Human Service guideline titled Guide for the Care and Use of Laboratory Animals, and with the Weatherall report (21). None of the animals, blood and tissue samples originated from the German Primate Center. The animals were fed monkey diet (Purina) supplemented daily with fresh fruit or vegetables and water *ad libitum*. Additional enrichment including the delivery of appropriate safe toys is provided and overseen by the Yerkes enrichment staff and animal health is monitored daily by the animal care staff and veterinary personnel, available 24/7. Monkeys are caged in socially compatible same sex pairs to facilitate social enhancement and well-being. Monkeys showing signs of sustained weight loss, disease or distress are subject to clinical diagnosis based on symptoms and then provided either standard dietary supplementation analgesics and/or chemotherapy. Monkeys whose symptoms cannot be alleviated using standard dietary supplementation, analgesics and/or chemotherapy were humanely euthanized using an overdose of barbiturates according to the guidelines of the American Veterinary Medical Association. The studies reported herein were performed under IACUC protocol #2001186 “Innate immunity in SIV infection” which was reviewed and approved by the Emory University IACUC. It has been assigned the IACUC protocol number “YER-2001186-082414GA.” The Yerkes National Primate Research Center has been fully accredited by the Association for Assessment and Accreditation of Laboratory Animal Care International since 1985. All experiments were reviewed and approved by the Emory institutional animal use and care as well as biosafety review Committees.

### SIV infection of rhesus macaques

**Figure 1:**
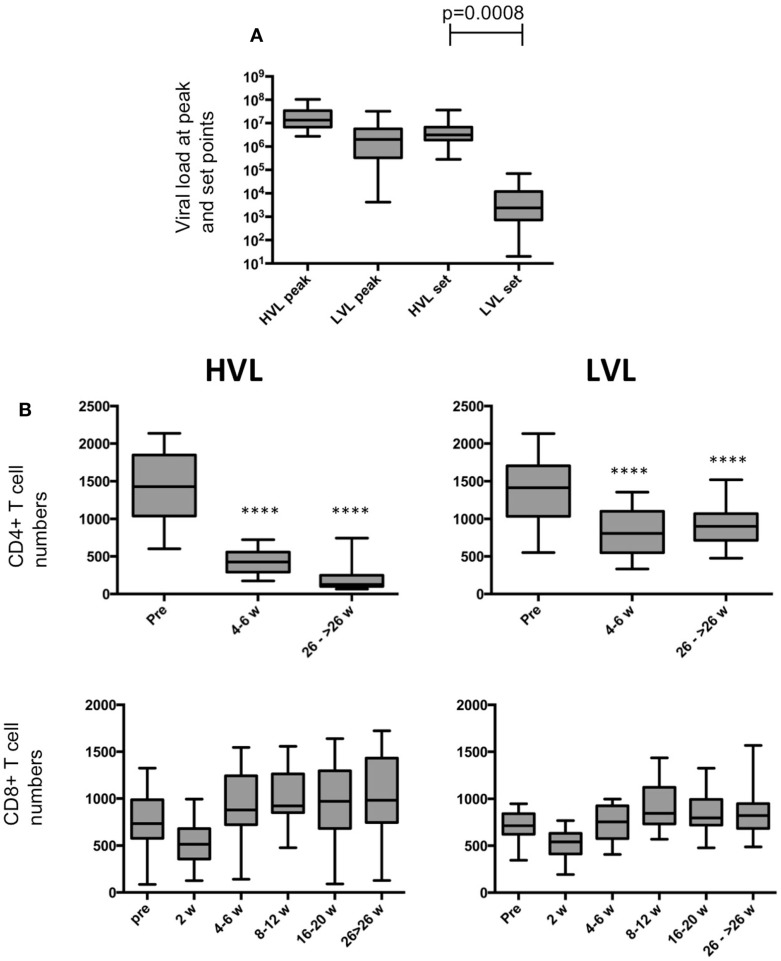
**Viral loads and T cell numbers**. **(A)** Plasma viral loads at peak and set point in HVL and LVL animals. Viral loads of HVL and LVL animals differ significantly at set point (*t*-test, *p* = 0.0008). **(B)** Box and whisker plots of CD4+ T cell and CD8+ T cell numbers during course of SIV infection in peripheral blood of HVL and LVL animals. T-tests were performed between the cell numbers of corresponding time points and were indicated as ****p < 0.0001.

**Figure 2:**
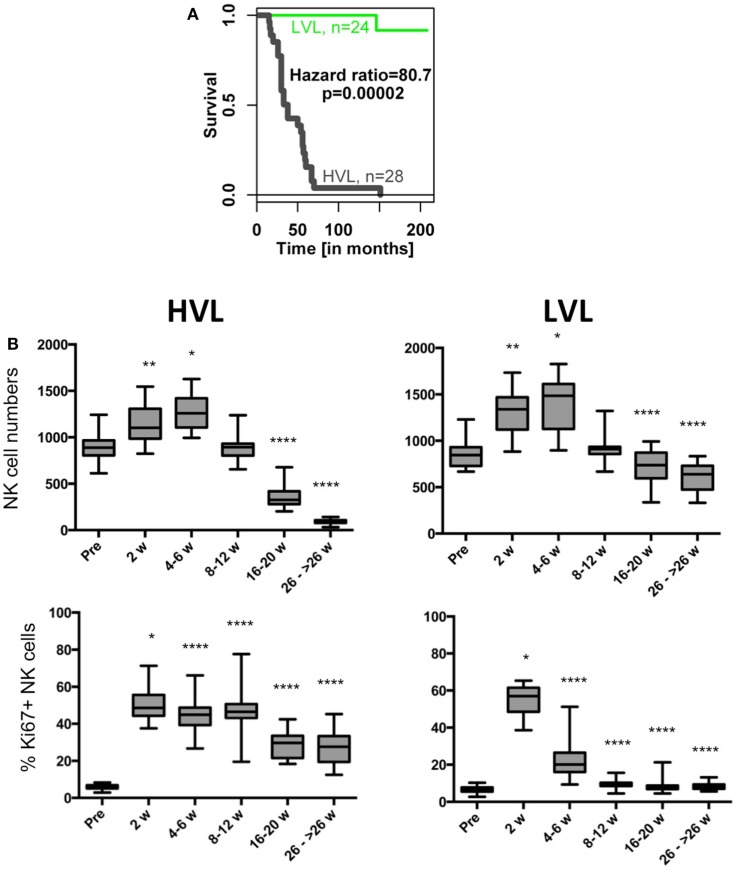
**Survival times as well as NK cell counts and percentage of Ki67-positive NK cells during the course of SIV infection in HVL and LVL animals**. **(A)** Kaplan–Meier survival curve of both strata. **(B)** Box and whisker plots of NK cell numbers (upper panel) and % Ki67-positive NK cells (lower panel). Sampling of cells was before infection (pre) and at indicated weeks after infection. *t*-Tests were performed between the cell numbers of corresponding time points and were indicated as *p < 0.05; **p < 0.01; ****p < 0.0001.

This study was conducted on a cohort of 52 juvenile to adult male rhesus macaques that were infected intravenously with 1000 TCID50 of either SIVmac251 (*n* = 5) or SIVmac239 (*n* = 47) grown in con-A blast cultures from normal rhesus macaques. Statistical analyses in this report results were fairly robust when excluding the five SIVmac251 infected animals (data not shown). On the other hand including these animals notably increased statistical power by increasing the sample size. Based on viral loads following achievement of stable set points (that correlated with absolute peripheral blood CD4+ T cell levels, and clinical characteristics including hemogram values, weight gain/loss and blood chemistries, see Table 1) the cohort was stratified into rhesus macaques with low plasma viral load (LVL (*n* = 24); <10^5^ mean ± S.D. viral copies/mL of plasma; slow progression to AIDS) and rhesus macaques with high plasma viral load (HVL (*n* = 28); >10^5^ mean ± SD viral copies/mL of plasma; rapid progression to AIDS). Figure 1 shows box and whiskers plots of viral loads at set point, indicating that the virus copy numbers are significantly different (*p* = 0.0008) and non-overlapping between the two strata, and Figure 2 underlines the distinctly different survival times between HVL and LVL. Blood samples from each monkey were obtained three times prior to SIV infection to derive average baseline absolute numbers of the various cell lineages. The monkeys were bled once per week post infection for 6–8 weeks and then biweekly for an additional 2 months and monthly thereafter to monitor plasma viral copy numbers and absolute numbers of lymphoid cell subsets. Cellular viral loads in aliquots of PBMC and colorectal biopsy tissue samples were monitored using a technique standardized in our lab that included negative and positive controls.

**Table 1:** **Overview of the macaque cohort (*n* = 52)**.

Outcome	LVL, *n* = 24			HVL, *n* = 28			
Virus^a^	SIVmac239, *n* = 22	SIVmac251, *n* = 2		SIVmac239, *n* = 25	SIVmac251, *n* = 3		
	Summary statistic	Individual data		Summary statistic	Individual data		
	Median (range)	mm34	mm38	Median (range)	mm19	mm25	mm27
Sex	100% male	Male	Male	100% male	Male	Male	Male
*Age at pre-infection (days)*							
	1492 (median)	1680	2061	1555 (median)	1695	1810	1801
	658–4549 (min–max)			1024–4570 (min–max)			
*Intravenously injected viral dose:* 1000 TCID50 per animal							
*CD4^+^ T cell number in peripheral blood*							
At pre-infection	1364 (552–2018)	2134	1567	1350(601–2121)	1884	1887	2139
At ≥26 weeks p.i.	918.0 (477–1519)	667	612	150.5 (66–742)	109	73	109
*Viral load in peripheral blood*							
At peak	2.2 × 10^6^ (4.2 × 10^3^–32.6 × 10^6^)	0.3 × 10^6^	1.1 × 10^6^	15.0 × 10^6^ (2.8 × 10^6^–104.0 × 10^6^)	10.8 × 10^6^	21.0 × 10^6^	6.7 × 10^6^
At set point	2.4 × 10^3^ (20–70.2 × 10^3^)	4.0 × 10^3^	1.5 × 10^3^	3.3 × 10^6^ (0.3 × 10^6^–36.8 × 10^6^)	3.0 × 10^6^	4.0 × 10^6^	2.0 × 10^6^
**Outcomes of interest**							
*Survival time (in month, censored times indicated by >)*							
Median	>200 month	>73	>156	38 month	151	>14	54
First event	146 month			15 month			
Last event	–			70 month			
# Events	1 event			26 events		
*Viral load in gastro-intestinal tissue*							
	9 (3–27)	Missing	6	22 (8–92)	19	46	27
*Number of NK cells*							
At pre-infection	860.5 (668–1229)	831	729	884.0 (612–1242)	933	893	789
≥26 weeks p.i.	639.5 (348–834)	332	673	94.0 (32–143)	87	124	143
*%Ki67+ NK cells*							
At pre-infection	6.7 (4.2–10.3)	5.4	2.7	5.8 (2.9–8.3)	7.3	5.6	7.3
8–12 weeks p.i.	9.3 (4.5–15.6)	9.3	8.4	46.7 (29.6–77.6)	45.6	35.6	19.5
*%CD107a-positive NK cells*							
Pre-infection	18.3 (4.9–37.2)	Missing	22.5	23.7 (6.9–42.8)	32.7	13.9	19.3
Acute infection	18.6 (12.3–27.6)	Missing	15.4	16.8 (7.2–33.9)	13.2	14.3	6.9
Chronic infection	16.2 (8.1–30.1)	Missing	16.9	8.0 (0.1–24.6)	6.9	2.1	0.4

*While aminals were comparable at pre-infection, the cohort separated into two distinct subgroups (LVL, HVL) regarding set point viral loads. All other clinical parameters consistently indicate slow (LVL) or fast (HVL) disease progression*.

*^a^Virus strain did not associate with infection outcome (HVL outcome: *p* = 1.00, survival time: *p* = 0.591)*.

### Specimen collection, mononuclear cells isolation, virus quantitation

Peripheral blood mononuclear cells (PBMC) were isolated from heparinized whole blood using standard Ficoll-Hypaque centrifugation procedure. The cell count and cell viability were performed using the trypan blue dye technique. The cell concentrations were adjusted to 1 × 10^6^ cells/mL in complete RPMI1640 media. Aliquots of EDTA plasma were obtained prior to and at varying intervals following SIV infection and sent on dry ice to Siemens Laboratories (Siemens, Berkeley, CA, USA) for quantitating plasma SIV levels using the branched DNA assay. Data obtained are illustrated as plasma VL per milliliter of plasma. For the quantitation of pro-viral DNA load, aliquots of the unfractionated PBMCs and cells obtained from the colorectal gut biopsy tissues collected at various times post infection were used to isolate total genomic DNA using the Qiagen DNAeasy kit (Qiagen, Valencia, CA, USA). For purposes of brevity, only the data for pro-viral DNA levels on cells isolated from rectal biopsy samples obtained during viral load set point (week 8 p.i.) are shown. An aliquot of the genomic DNA isolated was used to quantitate the amount of DNA per sample, and the number of viral copies per nanogram of DNA was determined using our standardized laboratory protocol for quantitation of SIV gag (22, 23). The SIV1C cell line that has a single copy of SIV DNA per cell was used as a control for the pro-viral DNA analysis. The sensitivity of the assay was one infected cell per million. The quantitation of viral RNA was performed using RNA extraction, RNAse free primer pairs, and real-time PCR utilizing SYBR Green for iCycler kit as previously described (24). The sensitivity of this assay was 10 viral copies/mL.

### Polychromatic flow cytometry and fluorochrome-conjugated monoclonal antibodies

Various fluorochrome-conjugated monoclonal antibodies (mAbs) were used in appropriate combinations to stain aliquots of the PBMCs. The mAbs utilized were purchased from BD Biosciences (San Jose, CA, USA) and included Alexa700-anti-CD3 (clone SP34-2), PerCP, Pac Blue or V450-conjugated anti-CD4 (clone L200), FITC or V500-anti-CD8 (clones SK1, RPA-T8), FITC-anti-CD14 (clone M5E2), PerCP-Cy5.5 or APC-Cy7-anti-CD16 (clone 3G8), APC-Cy7 or PeE-Cy7-anti-CD20 (clones 2H7, L27), PE-Cy7-anti-CD56 (clone NCAM16.2), APC-anti-CD123 (clone 7G3), PerCP-Cy5.5-anti-HLA-DR (clone L243), FITC or PE-conjugated Ki67 (clone B56), Alexa700 or PE-Cy5-anti-CD107a (clone H4A3). The PE-anti-NKG2A (clone Z199) was purchased from Beckman Coulter (Brea, CA, USA) and FITC-anti-human γδTCR (clone SA6.E9) from Invitrogen. Standard gating strategies were utilized to identify CD3+ CD4+ αβT cells, CD3+ CD8+ αβT cells, CD3+ γδT cells, CD3− CD20+ B cells, CD3− CD8− NKG2A− CD14+ monocytes, as well as CD3-CD8+ NKG2A+ NK cells and respective subsets based on CD16 and CD56 expression (25, 26). The cells were incubated with the appropriate combination of fluorochrome-conjugated antibodies for 30 min covered with aluminum foil and kept at 4°C. The cells were then washed twice with FACS buffer and resuspended in FACS buffer containing 2% paraformaldehyde and kept under aluminum foil until analyzed using a LSRII flow cytometer (BD Immunocytometry Division, Mountain View, CA, USA). A minimum of 100,000 events were examined per sample and data were analyzed using FlowJo software (TreeStar, Ashland, OR, USA). An aliquot of the blood sample was utilized to perform complete blood count and the values utilized to calculate absolute number of each cell lineage.

### NK cell function analysis

An aliquot of the PBMC sample to be analyzed for NK functional activity was co-cultured *in vitro* with HLA class I-devoid K562 or 721.221 cells at an effector/target ratio of 10:1 for 6 h at 37°C in a humidified 7% CO2 incubator in the presence of Brefeldin-A (5 ug/mL, Sigma-Aldrich), Monensin (6 ug/mL, Golgi-Stop, BD Biosciences) and anti-CD107a (clone LAMP1, BD Biosciences). Media throughout consisted of RPMI 1640 supplemented with 10% fetal calf serum, 2 mM l-glutamine, 100 units/mL penicillin and 100 ug/mL streptomycin. Each assay included an aliquot of the same PBMC cultured alone (negative control) and an aliquot of the same PBMC incubated with 1.25 ug/mL phorbol-12-myristate 13-acetate (PMA, Sigma-Aldrich) and 0.25 ug/mL ionomycin (Sigma Aldrich) as a positive control. Only experiments in which the negative control gave <10% value and >3-fold increase between the negative and positive control were considered valid. After the co-culture, the cells were stained for viability using the Aqua LIVE/DEAD (Invitrogen) and stained with anti-CD3, CD8 alpha, NKG2A, CD16 and CD56 antibodies. The cells were washed and resuspended in the FACS buffer and analyzed using the LSRII flow cytometer. A minimum of 100,000 events were analyzed and the frequency of the gated population of CD3−, CD8+, NKG2A+ CD16+ cells that were CD107a+ determined using FlowJo software (TreeStar, Ashland, OR, USA). Data obtained in the experimental sample – the negative control was calculated and utilized to illustrate NK cell functional activity.

### Next-generation sequencing of *MHC class I* and *KIR* gene transcripts

*MHC class I* and *KIR* gene transcripts were analyzed from PBMC cDNA samples using Roche/454 Titanium chemistry in a Roche/454 GS Junior sequencer (Roche Applied Science) according to previously published methods (18, 27). Amplicon library preparation, emPCR, bead recovery and sequencing were performed according to the manufacturer’s instructions. Pools of twelve samples were sequenced on a single PicoTiterPlate. The emPCR conditions for *MHC class I* and for *KIR* amplicon libraries were performed following the standard protocol instructions and recommendations for sequencing of long-length amplicon libraries (≥550 bp), respectively. The ratio of DNA and capture beads used for emPCR was in the order of 1–2 molecules per bead.

For each individual, separate library files were generated by sorting of sequencing reads with identical multiplex identifiers (MID). Primer sequences were cut off. Only reads with identical sequences that occurred at least five times were considered in order to reduce sequencing errors. The filtered reads were aligned against reference sequences of all known rhesus macaque *MHC class I* alleles (IPD MHC database non-human primates http://www.ebi.ac.uk/ipd/mhc/nhp/index.html) and rhesus macaque *KIR* database (kindly obtained from Dr. Libby Guethlein, Stanford University, USA) using BLAST. Perfectly matching reads were identified from *MHC class I* and *KIR* gene databases. In order to detect alleles that are not included in these databases, a maximum of two mismatches compared to the reference sequences were allowed. As forward and reverse reads were aligned separately, sequences with a maximum of four mismatches, over the full read length were included in the final preparation of *KIR* transcription profiles of each rhesus macaque. Since the region of the sequenced *KIR* transcripts of about 560 nt (623 nt with primer regions) is not fully covered by a single read, sufficient counts (≥5) of matching forward and reverse reads needed to be found. The overlap between forward and reverse reads covers around one third (180 nt) of the sequenced part of the *KIR* gene transcript. We also tested the *KIR* profile allowing four or six mismatches (i.e., 8 or 12 in forward + reverse), but this lead to ambiguous alignments for some *KIR* gene transcripts, i.e., matching of single reads to multiple *KIR* genes (data not shown). Frequencies of filtered forward and reverse reads were summed up and the relative contribution of each *KIR* transcript was compared to the total number of *KIR* transcripts identified in each individual. The *MHC class I* and *KIR* gene transcript sequencing data of the entire 52 rhesus macaques are shown in Tables S1 and S2 in Supplementary Material.

### Statistical analyses

The various statistical software programs used included SAS, R, and GraphPad Prism 6.

**Figure 3:**
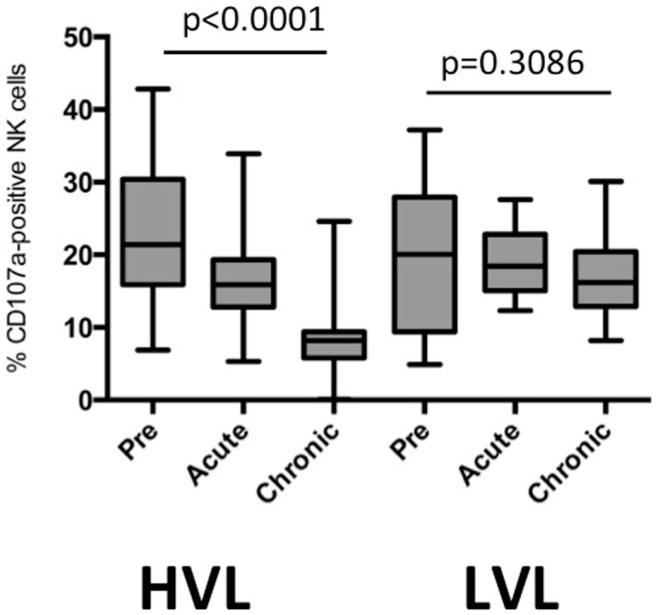
**Cytotoxic capacity of NK cells during different phases of SIV infection in HVL and LVL animals**. Degranulation after stimulation with MHC class I-deficient cells was determined as % CD107a-positive NK cells. Acute and chronic phases represent time points 2–4 weeks and >16 weeks after infection.

Time-course of NK cell numbers from 8–12 weeks p.i. to 26 to >26 weeks p.i. was analyzed by longitudinal linear regression, testing statistical interaction between covariates time and group (HVL, LVL) (see Figure 2). Difference of means of % Ki67 + NK cells at 8–12 weeks p.i. between HVL and LVL animals was examined by t-test; and the lytic function of NK cells was analyzed in HVL and LVL by repeated measures ANOVA (Figure 3, testing change of mean%CD107a-positive NK cells over time, i.e., from pre- to acute and chronic infection).

Target HVL outcome was analyzed by logistic regression, survival times by Cox regression, and NK cell number and corresponding functional data as well as viral loads in gut tissue by linear regression models, testing *KIR* main effects (Table 3, multiple or single *KIR* covariates as indicated) and *KIR*–*MHC class I* epistasis (see Table 4 and Figures 5 and 6; statistical interaction models contained a *KIR* and a *MHC-I* covariate, respectively). Furthermore, for the main target HVL outcome, we compared a multiple model (multi-*KIR*) with univariate analyses (single-*KIR*) (see Table 3, first columns). In contrast to single-*KIR* analyses, the multi-*KIR* model estimates mutually adjusted *KIR* main effects and accounts for correlations between *KIR* genes (*a priori* excluded were *KIR* genes that had small strata size or strong correlations with other *KIR* genes). In addition, the frequency of *KIR* transcript sequence reads at pre- and acute-infection (where NK cell numbers were high and comparable across HVL and LVL) was jointly analyzed in *KIR*-positive animals as longitudinal metric target by longitudinal rank-sum test (28) for association with HVL outcome (Table 3, last column). Rank-sum testing ensured test validity given that *KIR* transcript frequencies had skewed distributions. The rank-sum test resembles a heteroscedastic 1-way repeated measures ANOVA with factor HVL outcome on the longitudinal rank-order data of observed *KIR* transcript frequencies. Longitudinal analyses incorporated all animals that had any trait data (number of animals is indicated in Table 3; no omission of animals with incomplete follow-up and no imputation of missing values). We used SAS procedure *mixed* for longitudinal linear regression on% CD107a-positive NK cells and R package nparLD for longitudinal rank-sum testing of relative *KIR* transcript amounts (Table 3).

Association between *MHC class I* alleles and HVL outcome was tested by Fisher’s exact tests (see Table S3 in Supplementary Material). Finally, the probability to obtain K *p* values ≤ 0.05 for a family of M association tests with HVL outcome was estimated under the null hypothesis of no associations, based on 10,000 data replicates with randomly permuted HVL/LVL group assignment.

## Results

### NK cell numbers, proliferation, and activity during SIV infection

The cohort of rhesus macaques (*n* = 52) was experimentally infected with SIV (SIVmac) and subsequently stratified into animals with “low viral load” (LVL) and “high viral load” (HVL) based on viral load at set point (Figure 1A). Disease progressed much faster in HVL compared to LVL as indicated by absolute levels of blood CD4+ T cell counts and other clinical parameters (see Table 1 and Materials and Methods for details). The marked differences between HVL and LVL animals are further exemplified by a Kaplan–Meier survival curve (hazard ratio 80.7, *p* = 0.00002; Figure 2A). Table 1 shows a summary of the cohort and relevant data that are also displayed in Figures 1–3.

The disease course in HVL and LVL animals displays the typical marked (*p* < 0.0001) decline of CD4+ T cell counts in both strata (Figure 1B). Whereas the numbers of CD8+ T cells were rather similar (Figure 1B), we observed differences in blood NK cell numbers in LVL and HVL animals (Figure 2B). An increase of NK cell numbers during the acute phase of SIV infection (2–6 weeks p.i.) was followed by a steady decline in both strata. However, in contrast to LVL animals, HVL animals displayed a more marked decline in the number of NK cells during the chronic phase of infection by −30.6 per week (95% CI [−36.3, −24.9]; *p* < 0.0001). At 26 weeks p.i. and beyond, the mean absolute number of NK cells per μL blood in the HVL stratum was lower by −547.2 (95% CI [−608.6, −485.9]; *p* < 0.0001) compared to LVL animals. The proliferation of NK cells (% Ki67 +) peaked during the acute phase of infection (2 weeks p.i.) in both strata (Figure 2B). Interestingly, the percentage of Ki67 + NK cells rapidly declined in LVL animals and reached pre-infection levels at 8–12 weeks p.i. with a mean value of 9.7% (95% CI [8.7, 10.8]), whereas in contrast a significantly higher amount of proliferating NK cells was observed in HVL animals as long as 8–12 weeks p.i. (*t*-test, *p* < 0.0001) with a mean value of 46.4% (95% CI [42.3, 50.4]) Ki67+ NK cells that was sustained significantly (*p* < 0.0001) at high levels during the chronic phase of infection. Thus, blood NK cells in HVL animals had higher proliferation activity than in LVL animals particularly at time points 4–12 weeks p.i., a time interval when NK cell numbers of both strata are still comparable.

Next we determined the capacity of NK cells from LVL and HVL animals to lyse MHC class I-deficient cells in a degranulation assay using samples obtained at different time points post infection. The percentage of CD107a + NK cells during transition from pre-infection to acute and chronic phases declined in HVL animals (repeated measures ANOVA, *p* < 0.0001), but remained stable in LVL animals (*p* = 0.3086) (Figure 3).

Collectively, these data demonstrate that NK cells from animals with fast progression to AIDS and shorter survival times had sustained proliferation after SIV infection, but lost their lytic capability and constantly decreased in number following peak viral load.

### *KIR* heterogeneity in the rhesus macaque cohort

**Figure 4:**
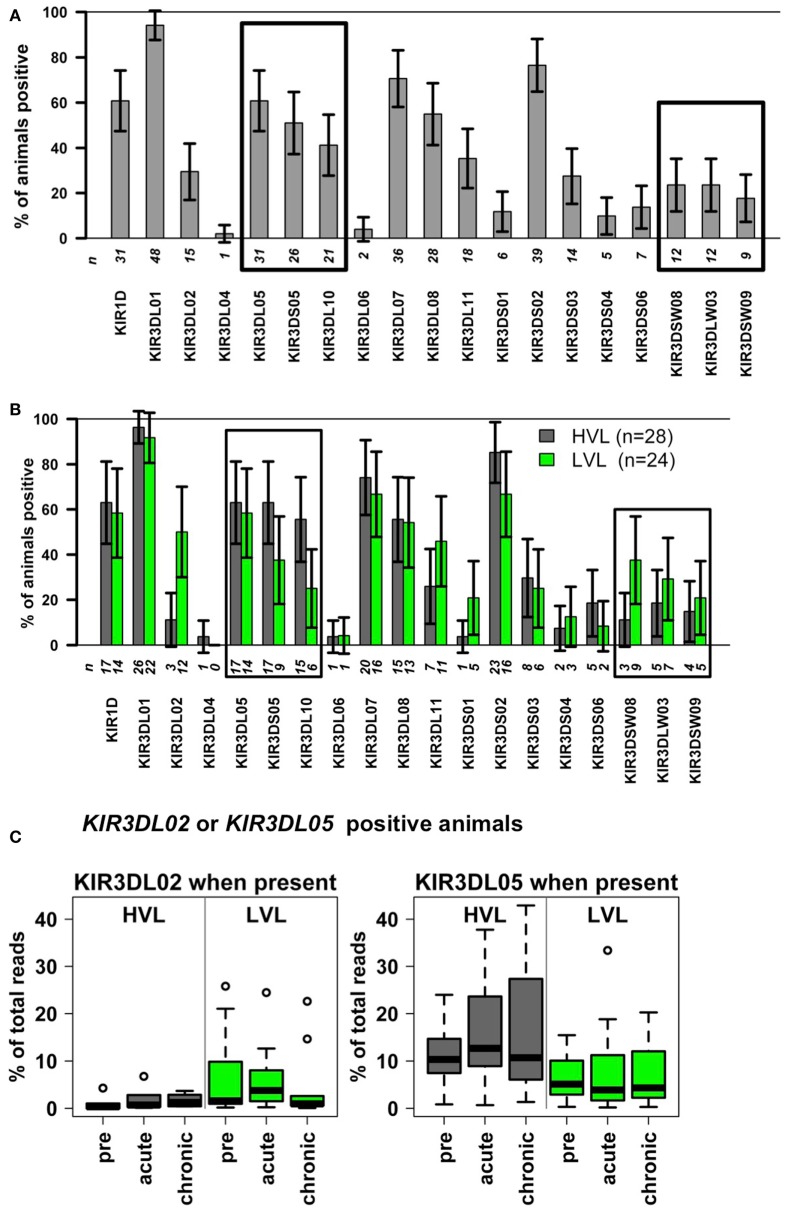
***KIR* genes in SIV infected rhesus macaques with slow (LVL, *n* = 24) and rapid (HVL, *n* = 28) progression to AIDS**. **(A,B)** Relative percentage of animals displaying the *KIR* transcript (bar height) with 95% confidence limits (error bars). The number *n* of animals transcribing the respective *KIR* gene is given below the bars. **(A)** Complete cohort of *n* = 52 rhesus macaques. Boxes indicate correlated *KIR* genes (*KIR3DL10*–*KIR3DS05*–*KIR3DL05*; *KIR3DSW08*–*KIR3DLW03*–*KIR3DSW09*; see Table 2). **(B)** Cohort stratified into HVL (*n* = 28) and LVL (*n* = 24) animals. **(C)** *KIR* transcript frequencies of animals transcribing the *KIR3DL02* or the *KIR3DL05* gene before (pre) and during different time points after infection (acute, chronic).

As KIR proteins essentially regulate the activity of NK cells, we determined the *KIR* gene transcripts by next-generation sequencing (Table S1 in Supplementary Material). Baseline values obtained pre-infection, showed similar NK cell numbers that ensured comparable transcript detection sensitivity across all animals. We found a similar gene diversity and frequency distribution of transcript-positive animals in our cohort as previously published by others (17, 18) for Indian rhesus macaques (Figure 4A). The most frequently transcribed *KIR* gene in our cohort was *KIR3DL01* (92%, *n* = 48), *KIR* genes particularly rarely transcribed were *KIR3DL04* (2%, *n* = 1), *KIR3DL06* (4%, *n* = 2), *KIR3DS01* (12%, *n* = 6), *KIR3DS04* (10%, *n* = 5), and *KIR3DS06* (13%, *n* = 7). Similar to the cohort analyzed by Moreland and co-workers (18), we detected no transcripts of the *KIR3DS07* gene in our cohort. Thus, the *KIR* gene diversity in our cohort is comparable to other cohorts.

### Association of *KIR* and *MHC CLASS I* polymorphisms with progression to AIDS

We initially tested whether the presence of individual *KIR* genes is correlated, for example due to close genetic linkage, which has hitherto not been determined for rhesus macaque *KIR* genes. Of particular interest for subsequent genetic association analyses with SIV infection outcome are strong correlations as well as distinctly different correlations in HVL compared to LVL. The latter may occur in the vicinity of functionally relevant genes when specific haplotypes associate with HVL outcome. As can be seen in Table 2, correlation between *KIR3DL05*–*KIR3DS05*–*KIR3DL10* and *KIR3DSW08*–*KIR3DLW03*–*KIR3DSW09* strongly suggests that these are neighboring genes (in the given order). Stronger correlation in HVL compared to LVL animals may indicate some functional relevance of these gene sets to infection outcome and differences in *KIR* haplotypes.

**Table 2:** **Correlation of *KIR* transcript presence at pre-infection**.

	Whole cohort (*n* = 52)		HVL animals (*n* = 28)		LVL animals (*n* = 24)	
	*KIR3DS05*	*KIR3DLW03*	*KIR3DS05*	*KIR3DLW03*	*KIR3DS05*	*KIR3DLW03*
*KIR3DL10*^a^	**0.74 (*p* = 5.2 × 10^−10^)**		**0.70 (*p* = 4.3 × 10^−5^)**		**0.75 (*p* = 2.9 × 10^−5^)**	
*KIR3DL05*^a^	0.42 (*p* = 0.0023)		**0.84 (*p* = 3.9 × 10^−8^)**		−0.04 (*p* = 0.84)	
*KIR3DSW08*^b^		**0.56 (*p* = 1.6 × 10^−5^)**		**0.74 (*p* = 9.6 × 10^−6^)**		0.45 (*p* = 0.027)
*KIR3DSW09*^b^		0.35 (*p* = 0.012)		0.61 (*p* = 8.0 × 10^−4^)		0.12 (*p* = 0.57)

*Displayed correlations (Pearson estimate, *p* value) were strong enough to have *p* ≤ 10^−3^ in HVL or LVL (significances in bold, Bonferroni: *p* ≤ 0.05/240 = 2.08 × 10^−4^ for testing pair wise correlations of 16 *KIRs* in HVL and LVL). *KIR3DL04* (2%), *KIR3DL06* (4%), *KIR3DL01* (92%) were not tested*.

*^a^*KIR3DL10* tended to correlate with *KIR3DL05* in the whole cohort (*r* = 0.35, *p* = 0.013) and in HVL (*r* = 0.55, *p* = 0.003) but not in LVL (*r* = 0.10, *p* = 0.65)*.

*^b^*KIR3DSW08*, *KIR3DSW09* were uncorrelated in the whole cohort (*r* = −0.01, *p* = 0.92) with opposing but non-significant tendencies in HVL (*r* = 0.18, *p* = 0.36) and LVL (*r* = −0.19, *p* = 0.39)*.

For *KIR* association analyses (Table 3) we converted levels of *KIR* transcript at pre-infection into binary variables that were subsequently used as covariate(s). Target traits for association analyses included HVL outcome (logistic regression), survival time (Cox regression), NK cell number and functional data (linear regressions). *KIR* effect estimates were mutually adjusted in a single multi-*KIR* model of HVL outcome, which was the main target (Table 3, first column, significances *p* ≤ 0.05 in bold; excluded were *KIRs* with small strata size, and *KIR3DS05* and *KIR3DLW03* because of strong correlations with neighboring *KIR* genes as shown in Table 2). All other association analyses in Table 3 employed single-*KIR* models. Furthermore, the frequency of *KIR* transcript sequence reads at pre- and acute-infection (where NK cell numbers were high and comparable across HVL and LVL) was jointly analyzed in *KIR*-positive animals as a longitudinal metric target for association with HVL outcome (Table 3, last column).

**Table 3:** ***KIR* association with HVL outcome, survival and NK cell function**.

Target trait	HVL outcome^a^				Survival^b^		Number of NK cells^c^		%Ki67 + NK cells^d^		%CD107a-positive NK cells^e^		Frequency of KIR transcript sequence reads^f^
**Binary covariate (s)**	***KIR* transcript presence at pre-infection**												**HVL outcome**
	**Multi-*KIR***		**Single-*KIR***		**Single-*KIR***		**Single-*KIR* At ≥ 26 weeks p.i**.		**Single-*KIR* At 8–12 weeks p.i**.		**Single-*KIR* Longitudinal time-course**		**Longitudinal level**
***KIR***	**OR**	**(*P*)**	**OR**	**(*P*)**	**HR**	**(*P*)**	**Δ**	**(*P*)**	**Δ**	**(*P*)**	**Δ**	**(*P*)**	**Rank-sum test (*P*)**

*1D*	0.60	(0.656)	1.21	(0.735)	1.12	(0.777)	−0.69	(0.993)	0.4	(0.947)	−1.0	(0.755)	(0.464)
*3DL01*	–	–	–	–	–	–	–	–	–	–	–	–	(0.358)
*3DL02*	**0.01**	**(0.0007)**	**0.13**	**(0.002)**	**0.24**	**(0.005)**	**220.7**	**(0.006)**	**−15.7**	**(0.005)**	**6.7**	**(0.033)**	**(0.033)**
*3DL04*	–	–	–	–	–	–	–	–	–	–	–	–	–
*3DL05*^g^	**0.09**	**(0.037)**	1.21	(0.735)	1.29	(0.536)	−48.7	(0.546)	1.6	(0.773)	1.1	(0.850)	**(0.022)**
*3DS05*^g^	–	–	2.83	(0.068)	1.84	(0.127)	−118.8	(0.128)	7.7	(0.174)	−4.7	(0.172)	(0.228)
*3DL10*^g^	0.98	(0.989)	**3.75**	**(0.025)**	1.96	(0.088)	**−180.5**	**(0.017)**	10.9	(0.056)	**−6.4**	**(0.035)**	(0.521)
*3DL06*	–	–	–	–	–	–	–	–	–	–	–	–	–
*3DL07*	5.26	(0.166)	1.43	(0.562)	1.46	(0.401)	−27.6	(0.750)	5.8	(0.330)	1.5	(0.831)	(0.291)
*3DL08*	17.24	(0.211)	1.06	(0.921)	1.11	(0.798)	15.9	(0.841)	0.5	(0.933)	−1.9	(0.456)	(0.150)
*3DL11*	0.47	(0.452)	0.41	(0.137)	0.47	(0.083)	116.4	(0.155)	−8.2	(0.165)	3.3	(0.410)	(0.688)
*3DS01*	–	–	0.15	(0.0504)	**0.20**	**(0.041)**	**243.9**	**(0.042)**	−15.5	(0.076)	5.1	(0.367)	(–)^f^
*3DS02*	**46.50**	**(0.008)**	2.88	(0.118)	**2.96**	**(0.043)**	−169.0	(0.065)	11.6	(0.056)	−3.8	(0.379)	(0.277)
*3DS03*	0.06	(0.238)	1.26	(0.711)	1.08	(0.861)	−53.1	(0.548)	3.3	(0.610)	1.3	(0.695)	(0.669)
*3DS04*	–	–	0.56	(0.541)	0.59	(0.448)	109.1	(0.410)	3.2	(0.741)	3.2	(0.638)	–
*3DS06*	–	–	2.50	(0.283)	2.07	(0.182)	−140.9	(0.216)	3.5	(0.656)	−3.4	(0.514)	–
*3DSW08*^g^	**0.10**	**(0.038)**	**0.21**	**(0.025**)	**0.29**	**(0.020)**	**222.8**	**(0.014)**	**−13.2**	**(0.025)**	1.6	(0.640)	(–)^f^
*3DLW03*^g^	–	–	0.55	(0.371)	0.62	(0.322)	90.3	(0.330)	−9.2	(0.127)	−1.8	(0.268)	(0.772)
*3DSW09*^g^	0.02	(0.084)	0.66	(0.574)	0.74	(0.570)	120.9	(0.240)	−8.0	(0.210)	−1.8	(0.353)	(0.732)

**P* values ≤0.05 in bold (significance in the multi-*KIR* model). Rare *KIR3DL04*, *KIR3DL06* (*n* ≤ 2 with this *KIR*) were not analyzed. The most frequent *KIR3DL01* (*n* = 4 without this *KIR*) was only analyzed for frequency of *KIR* transcript sequence reads*.

*^a^Logistic regression for target HVL outcome, with a single *KIR* or multiple *KIR* covariate(s): odds ratio (OR, <1 for protective *KIR*) and *p* value (*P*) (likelihood ratio test, χ^2^ statistic). ORs in the multi-*KIR* model are mutually adjusted within a single model [for *KIRs* with strata sizes ≥9 animals; excluding *3DS05*, *3DLW03* due to strong correlations (see Table 2)]*.

*^b^Cox proportional hazards model with a single *KIR* covariate: hazard ratio (HR, <1 for protective *KIR*) and *p* value (*P*) (likelihood ratio test, χ^2^ statistic)*.

*^c^Linear regression for target number of NK cells, with a single *KIR* covariate: *P* value (*P*) (*t* statistic) and *KIR* effect Δ (difference of trait means; Δ > 0 indicates a protective *KIR* that associates with higher NK cell numbers at ≥26 weeks p.i.)*.

*^d^Linear regression for target %Ki67+ NK cells, with a single *KIR* covariate: *P* value (*P*) (*t* statistic) and *KIR*-effect Δ (difference of trait means; Δ < 0 indicates a protective *KIR* that associates with moderated NK cell proliferation at 8–12 weeks p.i.)*.

*^e^Longitudinal target %CD107a-positive NK cells at pre-, acute, and chronic infection, analyzed by 1-way repeated measures ANOVA with factor *KIR* (*n* = 47 animals with data): *P* value (*P*) (ANOVA-F statistic for KIR × time interaction) and effect Δ in *KIR* carriers (difference between chronic and pre-infection trait means, compared to animals without *KIR* transcript). Note that in the cohort%CD107a-positive cells reduce over time (*p* < 0.0001) by −9.6%-points between pre- and chronic infection (see Figure 2). Thus, a *KIR* effect of 0 < Δ < 9.6 implies moderated loss-of-function (protective *KIR*), Δ < 0 enhanced loss-of-function (deleterious *KIR*)*.

*^f^Longitudinal target *KIR* transcript frequency at pre- and acute infection (where NK cell numbers are still high), analyzed by longitudinal rank-sum test (heteroscedastic 1-way repeated measures ANOVA with factor HVL outcome on longitudinal rank-order data of the target). Analyses (a–e) above comprised the whole cohort; this analysis (f) only *KIR positive* animals [see Figure 4B for subsample sizes; rare *KIR’s*, e.g., *KIR3DS01* (5 LVL, 1 HVL) and *KIR3DSW08* (9 LVL, 3 HVL) were not analyzed]. *P* values (*P*) for association of *KIR* transcript frequency with HVL outcome; *KIR3DL02* and *KIR3DL05* are illustrated in Figure 4C*.

*^g^*KIR3DL10*–*KIR3DS05*–*KIR3DL05* and *KIR3DSW08*–*KIR3DLW03*–*KIR3DSW09* are correlated (see Table 2). The particularly strongly correlated middle *KIR* was excluded from the multi-*KIR* model*.

The presence of transcripts for *KIR3DL02* and *KIR3DSW08* was associated significantly with LVL outcome (see Table 3: significant odds ratios =1 and Figure 4B). The observable weaker power of the *KIR3DSW08* gene is attributed to its low frequency. Additionally, a trend for the even rarer gene *KIR3DS01* to be protective was identified by the single-*KIR* model (Table 3, OR 0.15, *p* = 0.0504). *KIR3DS02* was detected as a risk gene for HVL outcome, especially when *KIR* correlations were accounted for in the multi-*KIR* model (Table 3; OR 46.5, *p* = 0.008). Furthermore, *KIR3DL10* associated with HVL outcome (Table 3, OR 3.75, *p* = 0.025) in single-*KIR* analysis; with a similar trend for correlated gene *KIR3DS05* (Table 3; OR 2.83, *p* = 0.068), whereas the multi-*KIR* model implicated presence of *KIR3DL05* to associate with LVL outcome (Table 3, OR 0.09, *p* = 0.037). This difference arises from the intricate correlation structure between *KIR3DL05*–*KIR3DS05*–*KIR3DL10* described above. The association of *KIR3DS05* and *KIR3DL10* with HVL outcome was underscored by subsequent epistasis analyses with *MHC class I* alleles (see below).

Table 3 further illustrates that the association of *KIR* genes with HVL outcome is mirrored in clinical (survival time) and functional (decline of blood NK cell numbers, sustained blood NK cell proliferation, and loss of blood NK cell lytic function) parameters. Indeed, transcription of the protective *KIR* genes *3DL02* and *3DSW08* is in both cases significantly associated with (1) longer survival time (*3DL02*: HR 0.24, *p* = 0.005; *3DSW08*: HR 0.29, *p* = 0.020), (2) higher numbers of blood NK cells (*3DL02*: + 220.7, *p* = 0.006; *3DSW08*: + 222.8, *p* = 0.014), and (3) lower numbers of proliferating NK cells (*3DL02*: −15.7%, *p* = 0.005; *3DSW08*: −13.2%, *p* = 0.025). Expression of *KIR3DL02* is further associated with better preservation of lytic capability (+6.7% CD107a positive, *p* = 0.033) and higher transcript frequencies in LVL animals compared to HVL animals (Table 3; Figure 4C, *p* = 0.033). Expression of the putatively protective *KIR3DS01* gene is associated with longer survival time (HR 0.20, *p* = 0.041) and higher NK cell numbers (+ 243.9, *p* = 0.042) and by trend also with less NK cell proliferation (−15.5%, *p* = 0.076) (Table 3), further underscoring the finding that *KIR3DS01* might be a protective *KIR* gene. Along the same line, transcription of the risk genes *KIR3DL10* and *KIR3DS02* is in both cases associated with shorter survival times (*KIR3S02*: HR 2.96, *p* = 0.043: *KIR3DL10:* borderline significance HR 1.96, *p* = 0.088), and with a decrease in NK cell numbers (*KIR3DL10*: −180.5, *p* = 0.017; *KIR3DS02*: borderline significance −169.0, *p* = 0.065). *KIR3DL10* is significantly associated with reduced lytic activity of NK cells (−6.4% CD107a-positive cells, *p* = 0.035) and both *KIR3DL10* and *KIR3DS02* show a trend for sustained proliferating NK cells at 8–12 weeks p.i. (both *p* = 0.056) (Table 3). Of note, all single-*KIR* effect estimates in Table 3 for *KIR3DS02* are remarkably similar to those of correlated *KIR* genes *3DL10*, *3DS05* across all analyses. Moreover, the most frequent *KIR* gene *KIR3DL05* in the correlated triplet (*3DL05*–*3DS05*–*3DL10*) and the highly frequent *KIR3DS02* tend to correlate (whole cohort: *r* = 0.41, *p* = 0.0031, HVL stratum: *r* = 0.54, *p* = 0.0034, LVL stratum: *r* = 0.30, *p* = 0.156, not significant after Bonferroni correction). Notably, *KIR3DL05* is associated with higher transcript frequencies in HVL animals compared to LVL animals (Table 3; Figure 4C, *p* = 0.022).

Furthermore, we determined the transcribed *MHC class I* alleles and identified 68 distinct *Mamu-A* and *Mamu-B* transcripts in the entire cohort of 52 rhesus macaques (see Table S2 for individual data and Table S3 for *MHC class I* gene/allele frequencies in Supplementary Material), with an average of 10 distinct transcripts per individual. We frequently observed coincidence of particular *MHC class I* transcripts (see Table S4 in Supplementary Material) that were previously found to co-occur on distinct *MHC class I* haplotypes (29, 30). It should be noted that rhesus macaque *MHC class I* haplotypes display extensive CNV of *Mamu-B* genes (29, 31–33). We found Mamu-A1*004 transcripts more frequently in the HVL than in the LVL stratum (OR 3.7, *p* = 0.029) (see Table S3 in Supplementary Material), and in accord with previous studies also Mamu-A1*001 (OR 0.4, *p* = 0.163) and the rare allele Mamu-B*008 (OR 0.0, *p* = 0.208) more frequently in the LVL than in the HVL stratum (not reaching significance for the latter two).

### Analyses of combinations of *kir* and *mhc class i* polymorphisms and progression to aids

KIR3DL1/3DS1 in combination with HLA-Bw4-80I allotypes (in particular HLA-B*57) are associated with slow progression to AIDS in human HIV infection (8, 9, 11, 34). Knowledge of the contribution of similar combinations of *KIR* and *MHC class I* alleles is currently not available in the model of SIVmac infection of rhesus macaques.

Sufficient strata sizes for epistasis analyses of *KIR* and *MHC class I* transcript presence were available for *MHC class I* alleles with cohort frequencies between 34.6 and 46.2% when combined with *KIR* genes with about 50% sample frequency. Of these *KIR* genes, the correlated putative risk genes *KIR3DS05*, *KIR3DL10* were the most interesting. Analyses of epistasis on HVL outcome (Table 4) consistently revealed higher power for *KIR3DS05* as well as deleterious effects of *KIR3DS05* and Mamu-A1*004. Epistasis test *p* values in Table 4 were not particularly significant. However, observing two (out of six) *p* ≤ 0.05 only had a probability *p* = 0.041 under the null-hypothesis of no epistasis and no genetic associations. Moreover, model estimates of *KIR3DS05* and *KIR3DL10* with Mamu-A1*001 were remarkably consistent. They indicate that the presence of *KIR3DS05* or *KIR3DL10* transcripts in Mamu-A1*001-negative animals associated with a 21.6-times higher HVL risk (*p* = 0.009). However, presence of Mamu-A1*001 transcripts annulled this detrimental effect of *KIR3DS05*, *KIR3DL10* (epistasis *p* ≤ 0.024; yielding an odds ratio of 1.1 for combined presence of Mamu-A1*001 with *KIR3DS05* or *KIR3DL10*).

**Table 4:** ***KIR3DS05*, *KIR3DL10* epistasis with *MHC class I* alleles on HVL risk**.

	*KIR3DS05*				*KIR3DL10*			

	*MHC class I*	*KIR3DS05*	Epistasis	*P*^a^	*MHC class I*	*KIR3DL10*	Epistasis	*P*^a^
*MHC class I*	OR_MHC_ (*p* value)	OR_KIR_ (*p* value)	OR_MHCxKIR_ (*p* value)		OR_MHC_ (*p* value)	OR_KIR_ (*p* value)	OR_MHCxKIR_ (*p* value)	
A1*001	1.50 (*p* = 0.622)	**21.60 (*p* = 0.009)**	**0.035 (*p* = 0.020)**	**0.041**	1.40 (*p* = 0.654)	**21.60 (*p* = 0.009)**	**0.036 (*p* = 0.024)**	**0.041**
A1*004	**11.00 (*p* = 0.015)**	**7.33 (*p* = 0.034)**	0.20 (*p* = 0.224)		3.64 (*p* = 0.101)	3.25 (*p* = 0.167)	1.10 (*p* = 0.942)	
B*012	0.50 (*p* = 0.471)	1.25 (*p* = 0.755)	10.00 (*p* = 0.087)		0.40 (*p* = 0.320)	1.20 (*p* = 0.811)	**25.00 (*p* = 0.036)**	
B*046	2.67 (*p* = 0.283)	**4.40 (*p* = 0.044)**	0.26 (*p* = 0.272)		1.30 (*p* = 0.745)	3.66 (*p* = 0.084)	1.03 (*p* = 0.984)	
B*057	**6.42 (*p* = 0.040)**	**6.60 (*p* = 0.028)**	0.17 (*p* = 0.153)		2.60 (*p* = 0.221)	4.33 (*p* = 0.089)	0.58 (*p* = 0.659)	
B*072	**10.29 (*p* = 0.047)**	**20.01 (*p* = 0.014)**	**0.054 (*p* = 0.043)**		3.00 (*p* = 0.179)	**8.00 (*p* = 0.029)**	0.29 (*p* = 0.330)	

*Displayed are all model term estimates (main effects and epistasis: odds ratio’s OR (*p* values), *p* ≤ 0.05 in bold) for twelve models of *KIR*–*MHC class I* polymorphism pairings. The two correlated *KIR* risk genes *KIR3DS05* and *KIR3DL10* were coded for transcript presence at pre-infection. The *MHC class I* alleles were coded for transcript presence, did not correlate significantly, and were frequent with sufficient strata sizes. The simultaneous risk estimates OR_MHC_, OR_KIR_, and OR_MHCxKIR_ quantify HVL risk of *MHC-I* in *KIR*-negative animals (OR_MHC_), HVL risk of *KIR* in *MHC-I*-negative animals (OR_KIR_) and epistatic effects for coincident presence of *KIR* and *MHC-I* (OR_MHCxKIR_). Animals with coincident *MHC-I* and *KIR* presence have the HVL risk OR_MHC_⋅OR_KIR_⋅OR_MHCxKIR_ compared to animals with coincident *MHC-I* and *KIR* absence*.

*^a^Probability that – under the null-hypothesis of no epistasis and no genetic associations – two or more *p* ≤ 0.05 for six epistasis tests of *MHC class I* alleles with a *KIR* gene. This probability was estimated based on 10,000 permutations of the HVL/LVL group assignment*.

Whereas Mamu-B*012 per se did not influence disease outcome according to our analysis (Table 4), there was a tendency of a higher HVL risk in animals with coincident presence of Mamu-B*012 with *KIR3DS05* or *KIR3DL10* transcripts (potential epistasis; note also the consistency of model estimates of Mamu-B*012 with *KIR3DS05* and *KIR3DL10*). Furthermore, it is of interest to note that Mamu-A1*004, Mamu-B*012, and Mamu-B*057 co-occur on *MHC class I* haplotypes that were previously reported for other rhesus macaques of Indian origin with rapid or intermediate SIV-disease progression (30).

**Figure 5:**
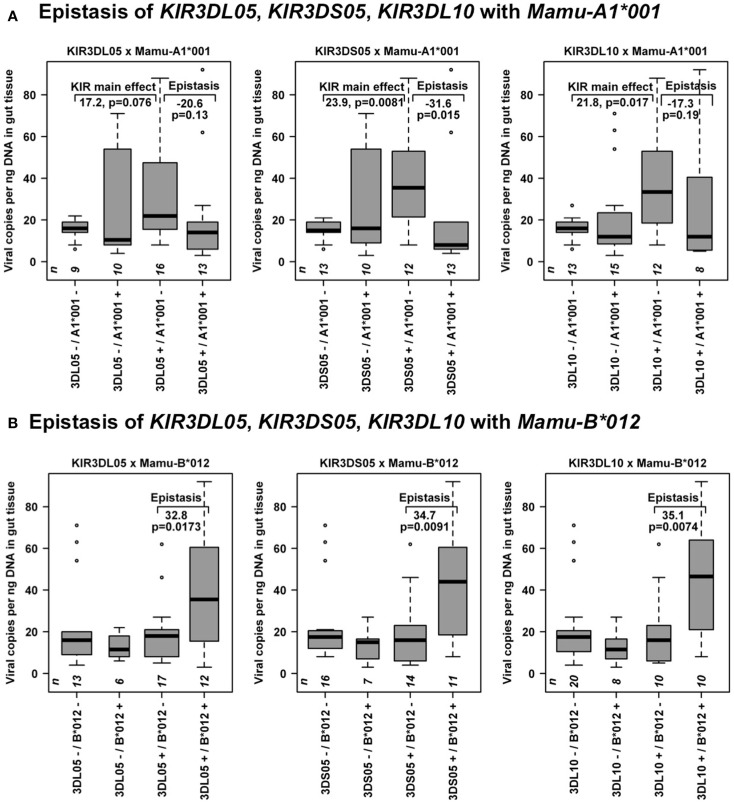
Epistasis of the correlated *KIR3DL05*–*KIR3DS05*–*KIR3DL10* genes with Mamu-A1*001 and Mamu-B*012 on viral copy numbers in GIT. The boxplots of viral copy numbers stratify for presence (+) and absence (−) of Mamu-A1*001 or *Mamu-B012* and the respective *KIR* (left panels: *KIR3DL05*, middle panels: *KIR3DS05*, right panels: *KIR3DL10*) transcripts. Sample sizes *n* are displayed at the bottom of the graphs. **(A)** Illustrated are the *KIR* main effects (directly observed in Mamu-A1*001-negative animals) and the epistatic effects of Mamu-A1*001 in *KIR3DL05-, KIR3DS05-*, or *KIR3DL10*-positive animals. **(B)** The epistasis model with Mamu-B*012 revealed consistent epistatic effects but no *KIR* main effects, suggesting that the *KIR* main effects (see Table 3 and this figure, part **A**) may be *marginal* effects from an epistasis with Mamu-B*012.

**Figure 6:**
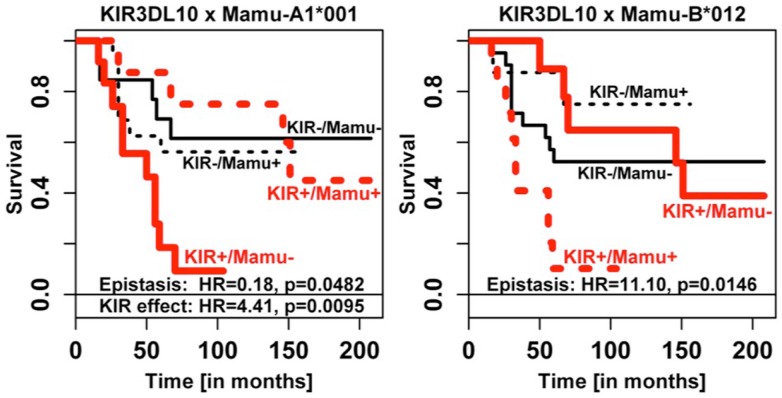
Epistasis of Mamu-A1*001 and Mamu-B*012 with *KIR3DL10* on survival time. The data for *KIR3DS05* are highly similar and are, therefore, not shown. Epistasis effect estimates on survival were consistent for *KIR3DL10* (see figure) and *KIR3DS05* (*KIR3DS05* × Mamu-A1*001: detrimental *KIR* effect HR 4.32, *p* = 0.0102, and epistasis HR 0.20, *p* = 0.0532; *KIR3DS05* × Mamu-B*012: epistasis HR 6.35, *p* = 0.0535). The more frequent *KIR3DL10* had higher statistical power.

While ORs had considerable estimation uncertainty (as is common in epistasis analyses), the epistasis of Mamu-A1*001 and Mamu-B*012 with *KIR3DS05*, and *KIR3DL10* on HVL outcome was also observed for viral copy numbers in GIT (Figure 5), and for survival time (Figure 6). In Mamu-A1*001-negative animals, viral copy numbers in GIT were particularly high in animals expressing *KIR3DS05* and *KIR3DL10*: 23.9 (*p* = 0.0081) or 21.8 (*p* = 0.017) more copies on average, respectively (Figure 5A). A similar trend can be seen in animals expressing the correlated *KIR3DL05* gene (17.2, *p* = 0.076). However, presence of Mamu-A1*001 transcripts is associated with an effect that is contrary to the *KIR3DL05*, *KIR3DS05*, and *KIR3DL10* main effects. In animals expressing *KIR3DS05*, the presence of Mamu-A1*001 transcripts was associated with decreased viral copies by −31.6 on average (*p* = 0.015). Similar trends were seen in animals expressing *KIR3DL10* or *KIR3DL05*, where the presence of Mamu-A1*001 transcripts coincided with decreased viral copy numbers by −17.3 (*p* = 0.19) or −20.6 (*p* = 0.13) on average (Figure 5A). Coincident presence of Mamu-B*012 with *KIR3DL05*, *KIR3DS05* or *KIR3DL10* transcripts resulted in 32.8 (*p* = 0.0173), 34.7 (*p* = 0.0091) or 35.1 (*p* = 0.0074) more viral copies on average, respectively (Figure 5B), confirming the suspected epistasis. Interestingly, no *KIR* main effects were seen in the epistasis models with Mamu-B*012 (Figure 5B). This suggests that the *KIR3DS05*, *KIR3DL10* main effects in the single-*KIR* analyses in Table 3 as well as in Figure 5A may be *marginal* effects from an epistasis with Mamu-B*012.

Figure 6 illustrates the fact that the presence of *KIR3DL10* transcripts when combined with lack of Mamu-A1*001 or with presence of Mamu-B*012 transcripts also led to shorter survival times, confirming the epistasis. Highly similar results were obtained for the correlated but the more rare *KIR3DS05* gene and for *KIR3DL05* (not shown), albeit with weaker power.

## Discussion

Our understanding of the biology of NK cells continues to evolve. Besides its killer function, data supporting the ability of cells within this lineage to become educated, acquire memory and participate in a number of immune regulatory function are accumulating (35–39). Consequently, the role of NK cells in HIV-1 infection and its corresponding non-human primate model has attracted much attention in AIDS research (20, 34, 40, 41). Particular attention in human HIV-1 infection has been paid to genetic factors that modulate NK-cell function and susceptibility to disease. Key determinants of NK-cell activation are the highly polymorphic KIR receptors and their MHC class I ligands. While human KIR3DL1/S1 allotypes in combination with HLA-B allotypes carrying the serological Bw4 epitope with an isoleucine residue at position 80 (HLA-Bw4-80I) were identified as protective, such combinations were hitherto not known in the rhesus macaque SIV infection model.

Based on viral load at set point that correlated well with clinical course (time to the development of AIDS) and absolute levels of peripheral blood CD4+ T cells, we grouped rhesus macaques experimentally infected with SIVmac into “HVL” and “LVL” strata. In contrast to LVL animals that had a relatively benign disease course, we found a progressive loss of peripheral blood NK-cell numbers after peak viraemia in animals rapidly succumbing to SIV-mediated disease (HVL). Further distinguishing HVL from LVL animals is a considerably higher percentage of proliferating (% Ki67 +) peripheral blood NK cells in HVL animals as long as 8–12 weeks p.i. (corresponding roughly with viral set point) when NK cell numbers were still high and comparable in both strata. This “proliferating” phenotype of NK cells in HVL animals (Figure 2B), however, is not associated with increased cytotoxic activity as evidenced by decreased CD107a-positive NK cells (Figure 3), indicating dysfunction of NK cells. Dysfunctional NK cells have been reported in HIV-1-infected individuals (42–44) and in SIV-infected rhesus macaques (45). Recent studies highlighted an important role of NK cells in the regulation of CD4+ and CD8+ T cell subsets in various viral infections (38, 46, 47). For example depending on the viral titer, LCVM infection is lethal to mice, but results in viral clearance if NK cells are depleted (38) or NK-cell deficient mice are used (46). The regulatory role of NK cells is exerted via killing of CD4+ or CD8+ T cells, leading to failure of CD8+ T-cell support and CD8+ T-cell responses. Consequently, depletion of NK cells improves CD8+ T-cell killing of virus-infected cells, enhances T-cell immunity and reduces immune pathology. Depending on the virus, such killing of T cells is perforin dependent or is achieved indirectly via cytokines released by NK cells. The reduced degranulation of NK cells in the HVL animals leads us to hypothesize that the T-cell response is affected in the SIV-infected HVL animals via NK cells’ cytokine secretion. Indeed, it has been proposed that cytotoxic CD16 + NK cells have only a limited role in the control of SIV (48). According to our data, this regulation obviously preferentially affects the CD4+ as compared with the CD8+ T-cell subset (Figure 1) and might be particularly effective in HVL animals 4–12 weeks p.i., a time point when NK cell numbers are still high and about 50% of these NK cells are proliferating (activated) in contrast to only 10–20% NK cells in the LVL cohort.

Control of lentiviruses might be influenced by NK cells at the local viral entry site with an important contribution by a NK cell subset that is uniquely present in such mucosal tissues (49). Indeed, rhesus macaques infected intra-rectally or intra-vaginally with SIV_mac251_ showed some functional differences of mucosal and blood NK cells, including a higher lytic capacity (% CD107a expression) of mucosal NK cells (50). However, whether differences in KIR expression are evident and responsible for these phenomena is currently not known.

We hypothesized that the observed functional differences of NK cells and the different disease outcomes are due to *KIR* and *MHC class I* polymorphisms. However, comprehensive molecular typing of *KIR* and *MHC class I* polymorphisms at the DNA level is extremely complicated in rhesus macaques due to large expansions of both gene families and extensive copy-number variations as well as an absence of detailed genomic information of haplotypes (only single *KIR* and *MHC class I* haplotypes are completely sequenced). Therefore, we used published next-generation sequencing approaches to determine transcripts of both rhesus macaque gene families (18, 27) and analyzed the risk of HVL outcome. Our study identified the inhibitory *KIR3DL02* and the activating *KIR3DSW08* (and most probably *KIR3DS01*) genes as protective factors in the rhesus macaque SIV infection model (Table 3; Figure 4). The activating *KIR3DS02* gene was detected as a risk gene, but only when *KIR* correlations were accounted for (multi-*KIR* model, Table 3). Of note, *KIR3DS02* had a certain correlation with the cluster *KIR3DL05*–*KIR3DS05*–*KIR3DL10*, in particular with *KIR3DL05* (whole cohort: *r* = 0.41, *p* = 0.0031; HVL stratum: *r* = 0.54, *p* = 0.0034; LVL stratum: *r* = 0.30, *p* = 0.156; non-significant after Bonferroni correction), and was in all single *KIR* analysis consistent with this *KIR* gene cluster (Table 3). While no specific ligand has so far been reported for KIR3DL02, a suggested ligand for KIR3DSW08 is Mamu-A3*13 (51). In accord with this, the Mamu-A3*13 allele (formerly designated Mamu-A*13) was reported to be associated with longer survival times of SIV-infected rhesus macaques (52) and is present on *MHC* haplotypes that were associated with slow, but also intermediate and rapid SIV disease progression (30). Unfortunately, frequencies of *KIR3DL02* and *KIR3DSW08* in the cohort (see Figure 4B, Table S1 in Supplementary Material) were not high enough to include both in the epistasis analyses. In addition, we had to exclude rare *KIR* genes (*KIR3DL04, KIR3DL06*; *n* ≤ 2, ≤ 3.8%) or *KIR* genes with high frequency (*KIR3DL01*; *n* = 48, 92.3%). Nevertheless, as about half of the animals (46%) belong to the LVL group, rare or very frequent genes are not expected to be responsible for the observed differences between LVL and HVL animals. Of note, the relative amounts of *KIR* transcripts were higher in LVL for the *KIR3DL02* gene and higher in HVL animals for the *KIR3DL05* gene, respectively (Figure 4C), supporting the aforementioned findings. Although we cannot exclude that these *KIR* genes show increased transcription, a more likely explanation for this observation appears to be a higher percentage of cells expressing these *KIR* genes, probably due to clonal expansions (T cells) or education (NK cells). As we analyzed transcripts from PBMCs, this might pertain to both *KIR*-expressing NK and T cell subsets. Furthermore, the association of *KIR* genes with HVL outcome was mirrored in clinical (survival time) and functional (decline of blood NK cell numbers, sustained NK cell proliferation, and loss of NK cell lytic function) parameters.

How can these associations of *KIR* genes with SIV disease outcome be explained? In general, involvement of inhibitory KIR expressed on cytolytic cells (NK, CD8+ T, γδ T) may lower the cytolytic activity of these cells toward infected cells (negative effects), but reduced activity may also result in less immune pathology (positive effect). Similarly, involvement of activating KIR proteins may enhance killing of SIV-infected cells by such cytolytic cells (positive effect), but may also evoke immune pathology (negative effect). Thus, beneficial and detrimental effects can be found for both inhibitory and activating KIR proteins and presence/absence polymorphisms in the individual’s genotype certainly influence the outcome. In the case of SIV infection we identified genes associated with a beneficial (*KIR3DL02*, *KIR3DSW08*) and others with a detrimental (*KIR3DL05*, *KIR3DL10*, *KIR3DS02*) outcome of infection.

In (uninfected) rhesus macaques, we previously identified KIR expression on 1–3% of CD4+ T cells, 4–25% of αβ CD8+ T cells, and 9–58% of γδ T cells (26). The role of KIR expression on T cell subsets is not as elaborated as for NK cells. It was previously shown that expression of inhibitory KIR on tumor-infiltrating human CD8+ T cells was associated with decreased effector functions such as interruption of TCR-mediated signaling and failure to rearrange the actin cytoskeleton (53). KIR expression is particularly seen in memory CD8+ T cells, but the exact role of KIR proteins in the formation of these cells is not known.

As strong linkage disequilibrium of *KIR* genes can perturb identification of corresponding risk genes in disease association studies, we analyzed coincidence of *KIR* gene transcripts and analyzed respective correlations. Indeed, *KIR3DL05*, *KIR3DS05*, and *KIR3DL10* as well as *KIR3DSW08*, *KIR3DLW03*, and *KIR3DSW09* correlated, respectively (Table 2). These correlations are most likely due to underlying haplotypes. Unfortunately, this assumption could not be tested as only a single rhesus macaque *KIR* haplotype is completely sequenced (54). Yet, these correlations complicated the analyses for *KIR3DL05*–*KIR3DS05*–*KIR3DL10* as none of these genes show consistent significant associations in all analyses such as for example *KIR3DL02*. However, for each of the various parameters tested at least one member of this *KIR* gene cluster shows significant (or borderline significance) association, indicating that cluster *3DL05*–*3DS05*–*3DL10* contains a risk *KIR* gene. Support for this assumption comes from epistasis analyses of *KIR* with *MHC class I* alleles. *KIR3DS05* and *KIR3DL10* only increased the risk of HVL outcome when Mamu-A1*001 was absent (odds ratio 21.6), but not when Mamu-A1*001 was present (odds ratio 1.1). This finding is particularly interesting as Mamu-A1*001 is a well-known protective MHC class I allotype (55, 56) and a ligand of KIR3DL05 (51, 57) and KIR3DS05 (51). A summary of the known KIR and MHC class I interactions is shown in Table 5. As Mamu-A1*001 is a well-known protective allele, the observed protective effect in epistasis analysis might also be attributed to this *MHC class I* allele only and not to epistasis with *KIR* genes. However, this appears rather unlikely given that (1) 35.7% (10/28) of the HVL animals indeed express Mamu-A1*001 (Table S2 in Supplementary Material) and are not protected, and (2) the mere presence of this *MHC class I* allele does not protect from high gut viral load (Figure 5A, compare for example 3DS05-/A1*001− with 3DS05-/A1*001+). Furthermore, statistical epistasis tests do not become significant because of consistent variant (*KIR* and *MHC*) effects but rather when such variant effects are statistically not independent (i.e., not additive for gut viral load). Thus, Mamu-A1*001 does not have a consistent protective effect across *KIR* strata and its effect obviously depends on epistasis with *KIR* genes. Further, the combined presence of Mamu-B*012 and *KIR3DS05* (OR 10.0, *p* = 0.087) or *KIR3DL10* (OR 25.0, *p* = 0.036) suggests a higher risk of HVL outcome. The biological significance of such epistasis is indicated by significant differences in gastro-intestinal viral load (Figure 5B) and in significantly shorter survival times of *KIR3DL10*-positive animals that lack Mamu-A1*001 or express Mamu-B*012 (Figure 6). Furthermore, epistasis analyses of HVL outcome, GIT viral load, and survival times suggest that *KIR3DS05* and *KIR3DL10* may not be detrimental *per se* for disease outcome, but only in combined presence with Mamu-B*012. In accord with our finding of Mamu-B*012 being a risk factor, this allele was particularly found on several haplotypes that were associated with rapid progression to AIDS (30). Some of the limitations of our analyses that are outlined above might be overcome in future SIV infection studies involving more animals and when complete *KIR* haplotypes are sequenced.

**Table 5:** **Known interactions of rhesus macaque KIR and MHC class I ligands**.

KIR	Interacting Mamu class I	Reference
KIR3DL01	Mamu-B with Bw4 motif	(58)
KIR3DLW03	Mamu-A1 alleles *001, *008; *011	(51)
KIR3DL05	Mamu-A1 alleles *001, *002; Mamu-A3*13	(51, 57)
KIR3DL11	Mamu-A1*008	(51)
KIR3DS05	Mamu-A1 alleles *001, *011	(51)

In a previous report Hellmann and colleagues (20) used Mamu-A1*001-negative SIV-infected rhesus macaques and found that variations in copy numbers of activating *KIR* genes associated with control of SIV replication. Yet, the individual gene(s) underlying this control were not further specified. In accord with the data of Hellmann and colleagues, we identified the activating *KIR3DSW08* gene (and most likely *KIR3DS01* as well) as protective in our cohort. The identification of the activating *KIR3DS05* as a risk factor in combination with Mamu-B*12 underscores the necessity to study associations at the level of individual genes as well as epistasis of the involved genes.

We conclude that a comprehensive survey of genetic factors that influence SIV-infection outcome needs both *MHC class I* and *KIR* genes in addition to other known anti-viral factors such as *TRIM5*. Such detailed knowledge is important when analyzing respective data of SIV-infected rhesus macaques to exclude any potential bias introduced by these disease-relevant genetic factors.

## Conflict of Interest Statement

The authors declare that the research was conducted in the absence of any commercial or financial relationships that could be construed as a potential conflict of interest.

## Supplementary Material

The Supplementary Material for this article can be found online at http://www.frontiersin.org/journal/10.3389/fimmu.2014.00600/abstract
